# Study of Antidepressant-Like Effects of Albiflorin and Paeoniflorin Through Metabolomics From the Perspective of Cancer-Related Depression

**DOI:** 10.3389/fneur.2022.828612

**Published:** 2022-07-08

**Authors:** Danping Zhao, Jianjun Zhang, Yingli Zhu, Cheng He, Wenting Fei, Na Yue, Chenglong Wang, Linyuan Wang

**Affiliations:** ^1^School of Chinese Materia Medica, Beijing University of Chinese Medicine, Beijing, China; ^2^School of Traditional Chinese Medicine, Beijing University of Chinese Medicine, Beijing, China; ^3^Beijing Institute of Traditional Chinese Medicine, Beijing University of Chinese Medicine, Beijing, China; ^4^Ethnic Medicine Characteristic Diagnosis and Treatment Center, Guangxi International Zhuang Medicine Hospital, Nanning, China

**Keywords:** albiflorin, paeoniflorin, depression, irradiation, metabonomics, mechanisms

## Abstract

Mental health has become a new challenge in cancer treatment, with a high prevalence of depression in patients with cancer. Albiflorin (AF) and paeoniflorinn (PF) are isomers extracted from the root of *Paeoniae Radix* Alba (Baishao in Chinese), belonging to the monoterpene glycosides, and multiple studies have been conducted on their antidepression and anti-cancer effects. However, the effects of AF and PF on cancer-related depression are unclear. Therefore, the current study aims to investigate whether the two isomers are able to exert antidepressant-like effects and understand the underlying mechanisms in a rat model, established by combining irradiation with chronic restraint stress and solitary confinement. Our results demonstrate a significant regulation of AF and PF in the pharmacodynamic index, including the peripheral blood, organ index, behavioral traits, and HPA axis, relative to control rats. In serum and cerebral cortex metabonomics analysis, AF and PF showed a significantly restorative trend in abnormal biomarkers and regulating ether lipid metabolism, alanine, aspartate, glutamate metabolism, tryptophan metabolism, carnitine metabolism, arachidonic acid metabolism, arginine and proline metabolism pathway. Eight potential biomarkers were further screened by means of receiver operating characteristic (ROC) analysis. The data indicate that AF and PF could effectively ameliorate a depression-like state in the model rats, and the mechanism may be associated with the regulation of the neuroendocrine immune system and disrupted metabolic pathways. Further experiments are warranted to comprehensively evaluate the antidepressant effects of AF and PF in cancer-related depression. This study provides a better insight into the action mechanisms of antidepression of TCM, and provides a new perspective for the therapy of cancer-related depression.

## Introduction

Cancer has a high morbidity and mortality worldwide: global cancer statistics for 36 cancers in 185 countries estimated 19.3 million new cancer cases and 10.0 million cancer deaths occurred in 2020 (1). Although several interventions, such as radiotherapy and chemotherapy, have improved the survival rates of cancer, new challenges have emerged, including psychological or mental health, with the prevalence of depression being high in patients with cancer (2, 3). A cross-sectional analysis of clinical data found that the prevalence of major depression ranged from 5.6 to 13.1% in several common cancers (4). Cancer-related psychological distress may limit or prevent the treatment course and recovery (5), reduce quality of life and increase health-care costs (6), while, even more problematically, some patients have suicidal thoughts (7). Health departments and cancer commissions in the US and the UK have all called for more attention to be paid to the depression in cancer patients (4). However, because of the lack of awareness among medical personnel and family caregivers, distress in cancer patients is frequently overlooked and under-treated (8). Furthermore, there are few effective pharmacotherapies (9, 10). Therefore, more attention and effective interventions are urgently needed.

*Paeoniae Radix* Alba (Baishao, PRA), derived from the dried root of *Paeonia lactiflora* Pall., is one of the most commonly used medicinal herbs in China (11), with the characteristics of nourishing the blood and smoothing the liver. Modern medical research has found that PRA has good analgesic, sedative and anti-inflammatory effects, and it is usually indicated for painful conditions, menstrual disorders and viral infections (12). Simultaneously, there are reports in the literature regarding the anticancer activities of PRA (13), and its major active constituents, paeoniflorin (PF) (14), and total glucosides of paeony (TGP) (15). However, few anticancer studies regarding albiflorin (AF), a monoterpene glycoside that is isomeric with PF (16), have been reported, which may be related to the difficulty found in its isolation and purification. The good news here is that our team has successfully isolated and purified AF and PF with high purity, and the method has also been obtained a Chinese patent certificate (ZL 201110184287.4). The hematopoietic effects of AF and PF were proven on radiotherapy-induced and chemotherapy-induced myelosuppression mice in our previous papers (17, 18), for which a Chinese patent certificate has also been obtained (ZL 201380078231.8). Concurrently, multiple studies have demonstrated that formulations of PRA have good antidepressant effects, including PF (19, 20), AF (21, 22), and Chinese prescription, such as Xiaoyaosan (23, 24), Sinisan (25), and Chaihu-Shu-Gan-San (26). Compared with the existing antidepressants in western medicine, which have a single mechanism of action, are ineffective and have obvious side effects in some patients, these formulations have the advantages of rapid action, high safety, and multi-target action (27), which may offer a new option for the treatment of depression.

**Figure 1:**
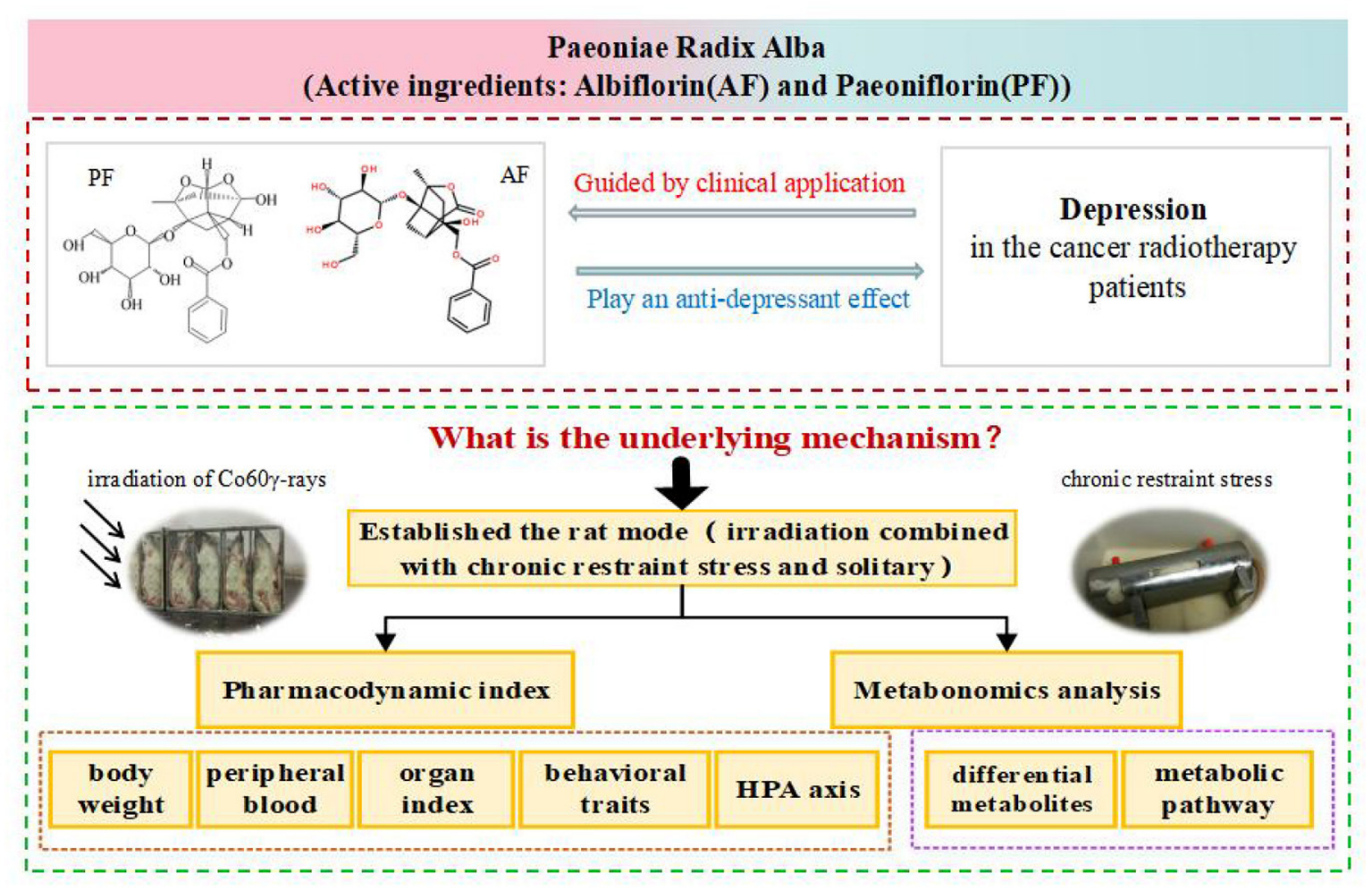
Research outline of the paper.

In this paper, taking advantage of previous research, we propose the hypothesis that AF and PF have antidepressant-like effects on depression in radiotherapy. To confirm our hypothesis, we established a rat model by combining irradiation with chronic restraint stress and solitary confinement, according to the state of depression in radiotherapy patients in a clinic, using the method previously confirmed by our team (28). Then, metabonomics was applied to investigate the therapeutic mechanisms (Figure 1). Our research provides an experimental basis for the clinical application of the antidepression effects of AF and PF in cancer radiotherapy patients.

## Materials and Methods

### Chemicals and Materials

MS-grade acetonitrile, methanol, isopropanol, water, ammonium acetate and foric acid were purchased from Fisher (Waltham, MA, USA). The blood routine test, adrenocorticotropic hormone, corticosterone and the hypothalamic kit were provided by Beijing Sinouk Institute of Biological Technology. *Paeoniae Radix* Alba (PRA) was purchased from YuLong Chinese medicine Company (Anguo, China) and authenticated before preparation by Prof. Zhang-Jian Jun, Beijing University of Chinese Medicine. The extract of PRA was extracted with water, and subject to vacuum concentrated drying (paeoniflorin =1.8% > 1.2% specified in Pharmacopeia of the People's Republic of China). Albiflorin (AF, purity = 98.6%) and paeoniflorin (PF, purity = 96.7%) were prepared in our library (patent number ZL 201110184287.4, China).

### Animal Experiment

A total of 40 male Sprague Dawley rats, weighing 200 ± 20 g, were housed for 5 days under controlled conditions before the experiments took place. The investigation was conducted in accordance with the Experimental Animal Ethics Committee of Beijing University of Chinese Medicine (No. BUCM-4-2017041807-2007). Throughout the study, all animals were kept under standard conditions regarding temperature (22 ± 2°C) and humidity (50 ± 10%), with a 12 h light/dark cycle. After several days of habituation, all the rats were randomly assigned to the following five groups: (1) normal group (N), (2) model group (M), (3) PRA group (PRA), (4) AF group (AF) and (5) PF group (PF), with each experimental group consisting of eight mice. With the exception of those from the control and model group, rats were intragastrically (i.g.) pretreated with the *Paeoniae Radix* Alba (2 g/kg), PF (30 mg/kg), and AF (30 mg/kg) for 21 days according to the previous study (16–18). The rats, with the exception of the normal group, were individually housed and received chronic restraint stress in the form of restricted movement for 3 h (8:00 a.m.−11:00 a.m.) for 21 days. On the 8th day, the model rats received total body irradiation of 3.5 Gy Co60γ-rays at a dose rate of 1.60 Gy. After irradiation, the administration of chronic restraint stress was continued for another 14 consecutive days.

### Sample Collection and Preparation

After the intragastric administration of PRA, PF and AF on the 21st day, all of the rats were anesthetized with a peritoneal injection of chloral hydrate, and blood samples were collected quickly in tubes from the abdominal aorta. Serum was obtained by means of centrifugation at 3,000 × g and 4°C for 10 min, and was frozen immediately at −80°C prior to analysis. For metabonomics analysis, serum was thawed at 4°C, and the samples were divided into two parts, namely the polar and non-polar parts. During the preparation of polar parts 100 μl of serum was aliquoted into a labeled 1.5 ml microcentrifuge tube, to which 300 μl of acetonitrile was added; this mixture was thoroughly mixed in a vortex mixer for 15 s, and the protein precipitate was pelleted in a centrifuge operating at 4°C and at 12,000 rpm for 10 min. We then transferred 100 μl of the supernatant into a 200 μl vial insert for analysis. In the preparation of non-polar parts, 100 μl of serum was aliquoted into a labeled 1.5 ml microcentrifuge tube and 600 μl chloroform/methanol (3:1) was added; the mixture was then ultrasonicated for 1 h, and then we added 100 μl of water, and mixed thoroughly. The mixture was then centrifuged at 4°C and at 12,000 rpm for 10 min. We took 300 μl of subnatant to dry, and then added 400 μl isopropyl alcohol/acetonitrile (1:1), and dissolved it using ultrasonication. This mixture was centrifuged at 12,000 rpm for 10 min, followed by transferring 100 μl of the supernatant into a 200 μl vial insert for analysis. Cerebral cortex tissue samples were weighed and ground, and 1 ml of methanol was added in order for the samples to mix well. The samples were thoroughly mixed in a vortex mixer for 30 min, and the protein precipitate was pelleted in a centrifuge operating at 4°C and at 12,000 rpm for 10 min. We then transferred 100 μl of supernatant into a 200 μl vial insert for analysis.

### Pharmacodynamic Index

#### Body Weight

During the experiment, the rats were weighed every week, and their weight was recorded.

#### Organ Index

After anesthesia and blood collection, the abdominal cavity was quickly opened and the thymus, spleen and adrenal gland were separated on ice and weighed. Thymus (spleen, adrenal gland) index = thymus (spleen, adrenal gland) weight/body weight.

#### Sucrose Preference Test

Rats were adapted to a 1% sucrose bottle in an individual cage for 24 h, then one bottle of 1% sucrose water and one bottle of tap water were given. The intake volumes were recorded, on days 0 and 21 of the experiment. Then, the sucrose consumption rate (%) = sucrose consumption/(water consumption + sucrose consumption) × 100%.

#### Open Field Test

On the 21st day of restraint, this test was carried out in an independent, quiet and dark room. Rats were placed in the center of an open box (80 cm × 80 cm × 40 cm), which had 25 equal squares (16 × 16 cm^2^) on the bottom with white lines, while the sides and bottom were black. The activity of the rat was observed and recorded within 3 min. The horizontal activity was based on the number of squares crossed by rat, and the vertical activity was based on the number of times the rat was upright (two forelimbs are more than 1 cm above the ground). After each rat completed the test, the open box was treated with alcohol to avoid leaving the smell of the previous rat.

#### Elevated Plus-Maze Test

On the 21st day of restraint, this test was carried out. The elevated plus-maze was composed of two opposing open arms (45 cm × 10 cm), two opposing closed arms (45 cm × 10 cm), and a central platform (10 cm × 10 cm) connecting the four arms, in the shape of a cross, and the inner wall and bottom were black, 50 cm above the ground. Rats were placed in the center of the central platform facing any open arm, and then we observed the rat's activity for 5 min, including: (1) the number of open arm entries, (2) the residence time in open arms, (3) the number of closed arm entries, (4) the residence time in closed arms. After one rat was measured, the maze was cleaned with a towel dipped in a low concentration of alcohol, and then the next test was carried out after the alcohol was dispersed.

#### Biochemical Indicators Examination

The contents of white blood cells (WBC), red blood cells (RBC) and hemoglobin (HGB) of peripheral blood were measured by an automatic blood cell analysis counter. Corticotropin-releasing hormone (CRH), adrenocorticotropic hormone (ACTH), and corticosterone (CORT) were measured using the rat enzyme linked immunosorbent assay (ELISA) kit according to the manufacturer's instructions.

### HPLC-LTQ/Orbitrap MS Analysis

The Thermo Scientific™ Dionex™ UltiMate™ 3000 Rapid Separation LC (RSLC) system performed UHPLC separations with a reversed phase C18 column or a hydrophilic interaction liquid chromatography column using the gradient conditions. RP separation for lipids: For C18 separation, mobile phase A was acetonitrile/water (60/40) and mobile phase B was isopropanol/ acetonitrile (90/10); both A and B contained 0.1% formic acid and 10 mmol/L ammonium acetate. The gradient conditions for reversed phase C18 separation were 0–2 min, 80%−70% A, 20%−30% B; 2–5 min, 70%−55% A, 30%−45% B; 5–6.5 min, 55%−40% A, 45%−60% B; 6.5–12 min, 40%−35% A, 60%−65% B; 12–14 min, 35%−15% A, 65%−85% B; 14–17.5 min, 15%−0% A, 85%−100% B; 17.5–18 min, 0%−0% A, 100%−100% B; 18–18.1 min, 0%−80% A, 100%−20% B; 18.1–19.5 min, 80%−80% A, 20%−20% B. The column was a HSS T3 column (2.1 × 100 mm, 1.8 μm, water) operated at 45°C. The flow rate was 300 μl/min, and the injection volume was 1 μl. HILIC separation: For HILIC separation, mobile phase A was acetonitrile and mobile phase B was water; both A and B contained 0.1% formic acid and 10 mmol/L ammonium acetate. The gradient conditions were 0–1 min, 95%−95% A, 5%−5% B; 1–7 min, 95%−50% A, 5%−50% B; 7–9 min, 50%−50% A, 50%−50% B; 9–9.1 min, 50%−95% A, 50%−5% B; 9.1–13 min, 95%−95% A, 5%−5% B. The column was a BEH Amide column (2.1 × 100 mm, 1.7 μm, waters) operated at 40°C. The flow rate was 300 μl/min, and the injection volume was 1 μl.

A Thermo Scientific™ Q Exactive™ hybrid quadrupole Orbitrap mass spectrometer equipped with a HESI-II probe was employed. The positive and negative HESI-II spray voltages were 3.7 and 3.5 kV, respectively, the heated capillary temperature was 320°C, the sheath gas pressure was 30 psi, the auxiliary gas setting was 10 psi, and the heated vaporizer temperature was 300°C. Both the sheath gas and the auxiliary gas were nitrogen. The collision gas was also nitrogen, at a pressure of 1.5 mTorr. The parameters of the full mass scan were as follows: a resolution of 70,000, an auto gain control target under 1 × 10^6^, a maximum isolation time of 50 ms, and an *m*/*z* range of 150–1,500. The LC-MS system was controlled using Xcalibur 2.2 SP1.48 software (Thermo Fisher Scientific), and data were collected and processed with the same software.

### Pattern Recognition Analysis and Data Processing

The raw data were analyzed using the Progenesis QI software for peak alignment, peak extraction and normalization. The extraction peak times of the non-polar part and polar part were 1–19 and 1–12 min, respectively, and the peak extraction intensity was limited to mode 3. Finally, to form a table detailing the retention time, mass/charge ratio and peak intensity, SIMCA14.1 software (Umetrics, Umea, Sweden) was used to perform principal component analysis (PCA) and partial least squares discriminate analysis (PLS-DA). According to the variable importance in the projection (VIP) of the Orthogonal PLS-DA (OPLS-DA) model (VIP > 1), we combined it with the *P*-value of the *t*-test (*P* < 0.05) to find the differentially expressed metabolites. At the same time, the structure of potential biomarkers was determined based on the fragmentation information of mass spectrometry and the accurate mass number, combined with HMDB, KEGG and other databases (error limit: 0.01 Da). Potential biomarkers were analyzed in MetaboAnalyst 4.0 (http://www.metaboanalyst.ca/), and screening impact value was greater than 0.1 pathways for potential related metabolic pathways. The Pearson correlation analysis between metabolites and pharmacodynamic index was also carried out on the MetaboAnalyst.

### Statistical Analysis

The data were analyzed using one-way analysis of variance (ANOVA) of IBM SPSS Statistics 20, followed by Tukey's test as a *post hoc* test. The values are expressed as the mean ± standard deviation (SD). Values of *P* < 0.05 were accepted as being statistically significant. All figures were created using GraphPad Prism^®^ software 7.0.

## Results

### Intervention Effects of Albiflorin and Paeoniflorin on the Pharmacodynamic Index on Model Rats

**Figure 2:**
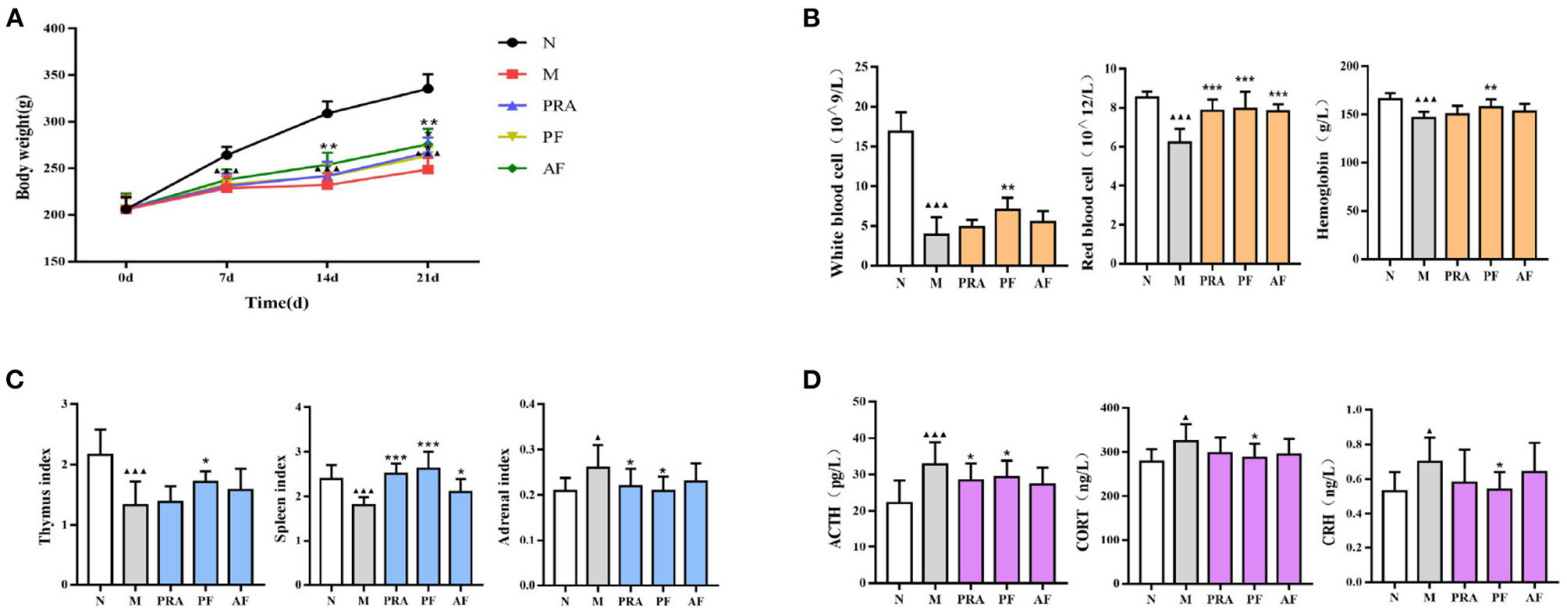
Changes in the pharmacodynamic index among groups. **(A)** The body weight of rats; **(B)** white blood cell, red blood cell and hemoglobin count in the peripheral blood; **(C)** thymus, spleen and adrenal organ index; **(D)** the HPA axis. The data are expressed as mean ± SD (*n* = 8). ^▴^*P* < 0.05, ^▴▴▴^*P* < 0.001, compared with the normal group; *P < 0.05, **P < 0.01, ***P < 0.001, compared with the model group. N, normal group; M, model group; PRA, Aqueous extract of *Paeoniae Radix* Alba group; PF, paeoniflorin group; AF, albiflorin group.

The pharmacodynamic index, including body weight, peripheral blood, organ index, and HPA axis, is displayed in Figure 2, and the behavioral experiment is shown in Tables 1, 2. With the prolongation of the modeling time, the rats became significantly lower in weight compared with the normal group (*P* < 0.001), and PRA, AF and PF significantly increased the body weight on the 21st day (*P* < 0.01, *P* < 0.001; Figure 2A).

**Table 1:** Changes in the sucrose preference and open field test among groups (*n* = 8, ±s).

**Group**	**Dosage/** **(mg/kg)**	**Sucrose preference**		**Open field test**	
		**0 day**	**21 days**	**Horizontal activity**	**Vertical activity**
N	—	85.37 ± 9.59	87.66 ± 3.99	52.62 ± 10.43	22.75 ± 7.14
M	—	85.03 ± 7.93	74.08 ± 8.73^▴▴▴^	35.62 ± 13.07^▴^	14.50 ± 6.59^▴▴^
PRA	2.0g/kg	85.65 ± 8.32	87.98 ± 5.28***	48.75 ± 13.09	18.12 ± 3.68
PF	30	84.38 ± 5.89	85.30 ± 5.72**	52.50 ± 10.12*	20.62 ± 5.44*
AF	30	86.55 ± 2.31	81.48 ± 8.01*	46.37 ± 19.63	21.00 ± 4.78*

^▴^ P < 0.05, ^▴▴^ P < 0.01, ^▴▴▴^ P < 0.001, compared with the normal group; *P < 0.05, **P < 0.01, ***P < 0.001, compared with the model group.

*N, normal group; M, model group; PRA, aqueous extract of Paeoniae Radix Alba group; PF, paeoniflorin group; AF, albiflorin group*.

**Table 2:** Changes in the elevated plus-maze test among groups (*n* = 8, ±s).

**Group**	**Dosage/** **(mg/kg)**	**Elevated plus-maze**			
		**Numbers of open-arm entry**	**Time of open-arm residence**	**Numbers of closed-arm entry**	**Time of closed-arm residence**
N	—	3.62 ± 1.50	25.91 ± 16.96	5.62 ± 2.72	151.49 ± 16.91
M	—	0.87 ± 1.12^▴▴▴^	5.71 ± 6.66^▴^	1.87 ± 1.12^▴▴^	172.16 ± 8.83^▴^
PRA	2.0g/kg	2.00 ± 1.69	14.64 ± 10.04	3.25 ± 1.98	148.29 ± 19.68*
PF	30	2.25 ± 1.03	33.59 ± 16.65**	3.75 ± 2.12	127.68 ± 22.34***
AF	30	1.75 ± 1.48	29.99 ± 19.57**	3.50 ± 2.39	141.11 ± 24.25**

^▴^ P < 0.05, ^▴▴^ P < 0.01, ^▴▴▴^ P < 0.001, compared with the normal group; *P < 0.05, **P < 0.01, ***P < 0.001, compared with the model group.

*N, normal group; M, model group; PRA, Aqueous extract of Paeoniae Radix Alba group; PF, paeoniflorin group; AF, albiflorin group*.

The WBC, RBC and HGB count in peripheral blood was used to evaluate body immunity, which in the model group was significantly decreased compared with the normal group (*P* < 0.001), while the WBC, RBC and HGB count in the PF group was significantly increased (*P* < 0.01, *P* < 0.001), and AF can increased RBC significantly (*P* < 0.001; Figure 2B).

The organ index can also be use to evaluate immunity. Compared with the normal group, the thymus index and the spleen index of the model group were significantly decreased (*P* < 0.001), and the adrenal index was significantly increased (*P* < 0.05). PRA significantly increased the spleen index and decreased the adrenal index (*P* < 0.001, *P* < 0.05). PF significantly increased the thymus and the spleen index (*P* < 0.001, *P* < 0.05), and decreased the adrenal index (*P* < 0.05), while AF increased the spleen index (*P* < 0.05; Figure 2C).

The HPA axis plays an important role in the pathogenesis of depression. In the results, compared with the normal group, the ACTH, CORT and CRH content in model group was significantly decreased (*P* < 0.01, *P* < 0.001), PF significantly increased these contents (*P* < 0.05), and the paeoniflorin group had an increased ACTH content (*P* < 0.05), while AF also demonstrated an increase (Figure 2D).

From the behavioral experiment, in which sugar water consumption, open field and elevated plus maze movement were restricted (*P* < 0.05, *P* < 0.01, *P* < 0.001), AF and PF can ameliorate behavioral deficits (*P* < 0.05, *P* < 0.01, *P* < 0.001). The pharmacodynamic index results indicated that AF and PF can antagonize depression and blood deficiency in model rats by regulating the neuroendocrine-immune network.

### Metabolic Profiles Analysis Based on PCA and OPLS-DA Analysis

**Figure 3:**
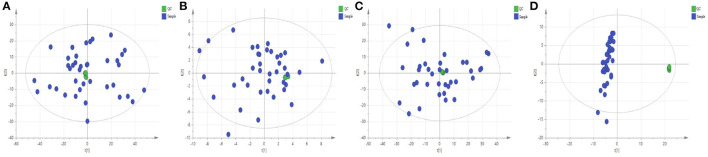
Quality control sample evaluation. **(A)** Positive mode of polar part of the serum; **(B)** Positive mode of non-polar part of the serum; **(C)** Positive mode of polar part of the cerebral cortex; **(D)** Positive mode of non-polar part of the cerebral cortex.

**Figure 4:**
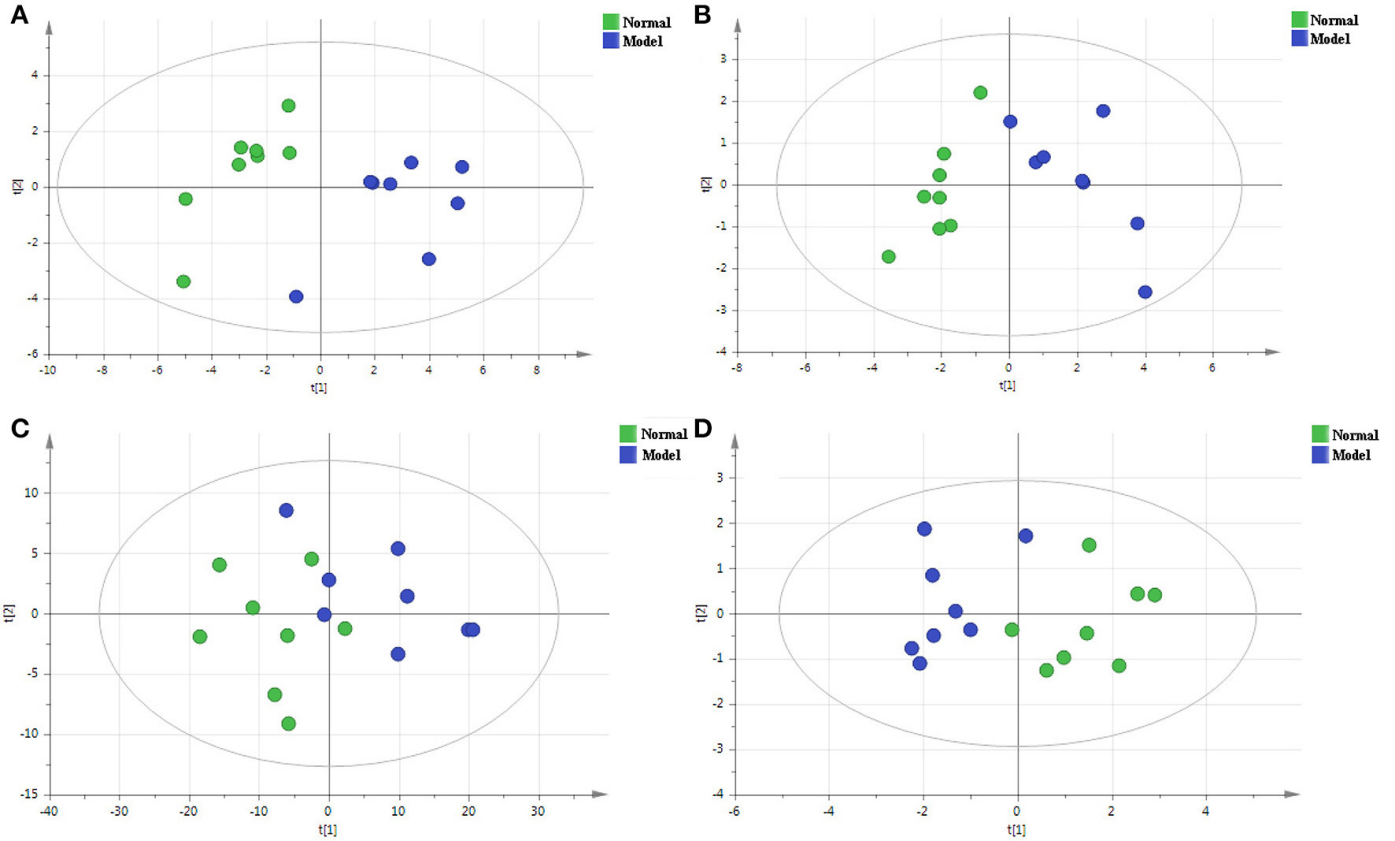
The PCA score plots of the normal group and the model group in the positive mode of the non-polar part **(A)** and the positive mode of the polar part **(B)** of the serum, and in the positive mode of the non-polar part **(C)** and the positive mode of polar part **(D)** of the cerebral cortex.

PCA is a non-supervisory model analysis method, which can classify the data according to their similarity, and can more truly reflect the differences between groups and identify the variation within groups. In PCA, R^2^X and Q^2^ are generally used to judge the quality of the model. R^2^X represents the interpretation rate of the model, and Q^2^ represents the predictable variables of the model (29). Generally, the greater the value of R^2^X and Q^2^, the more reliable the model is. The PCA analysis results of the QC sample and the normal group and the model group are shown in Figures 3, 4, respectively. As can be seen, the relative aggregation of QC samples demonstrated that the system had good repeatability, and the collected data can be further studied. The model group were obviously separated from the normal group. The R^2^X of the non-polar part was 0.736, and the R^2^X of the polar part was 0.614 in the serum sample, while in the cerebral cortex, the non-polar part's R^2^X was 0.734 and the polar part's R^2^X was 0.510, indicating that most of the data in the sample were used to establish the principal components, and the model was basically established successfully.

**Figure 5:**
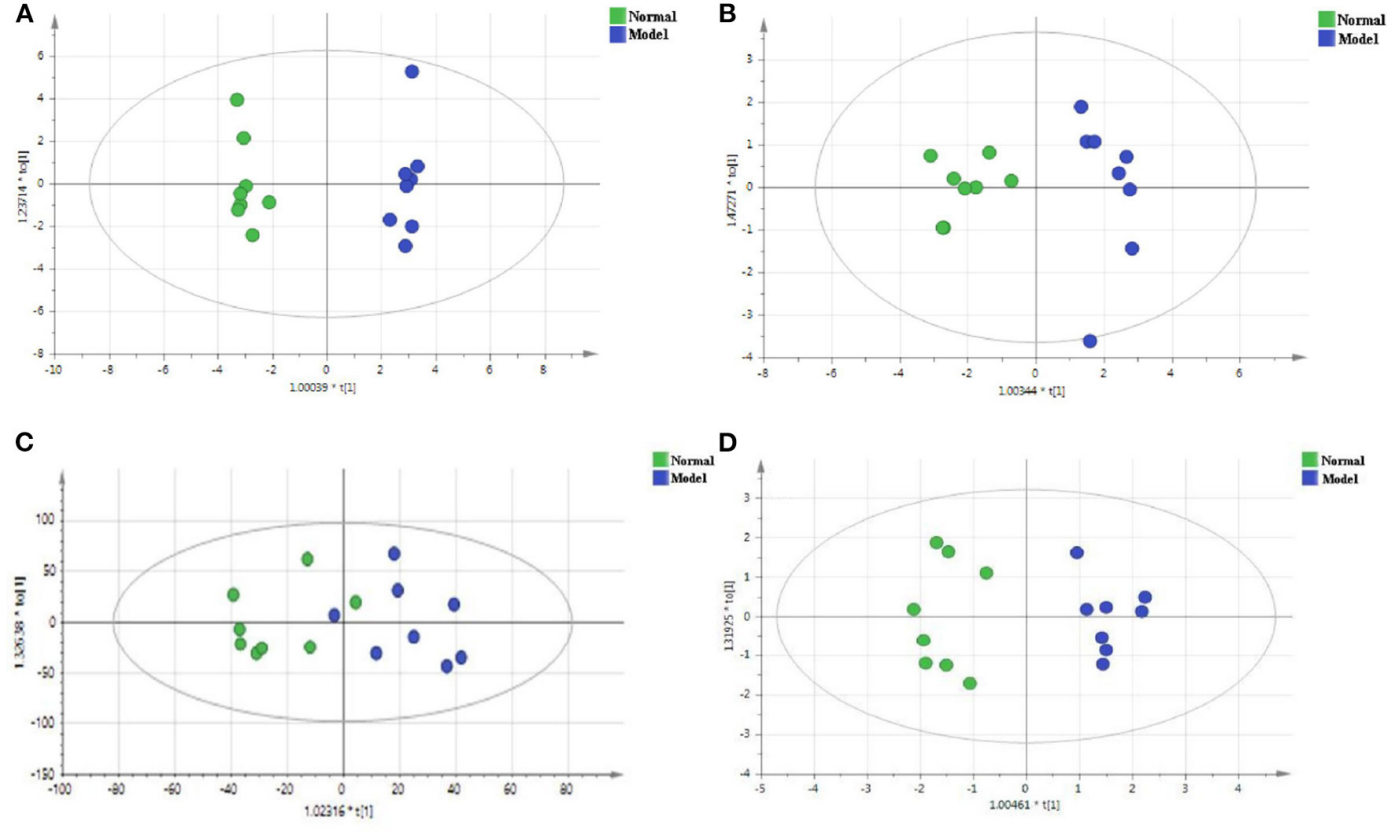
The OPLS-DA score plots of the normal group and the model group in the positive mode of the non-polar part **(A)** and the positive mode of the polar part **(B)** of the serum, and in the positive mode of the non-polar part **(C)** and the positive mode of the polar part **(D)** of the cerebral cortex.

**Figure 6:**
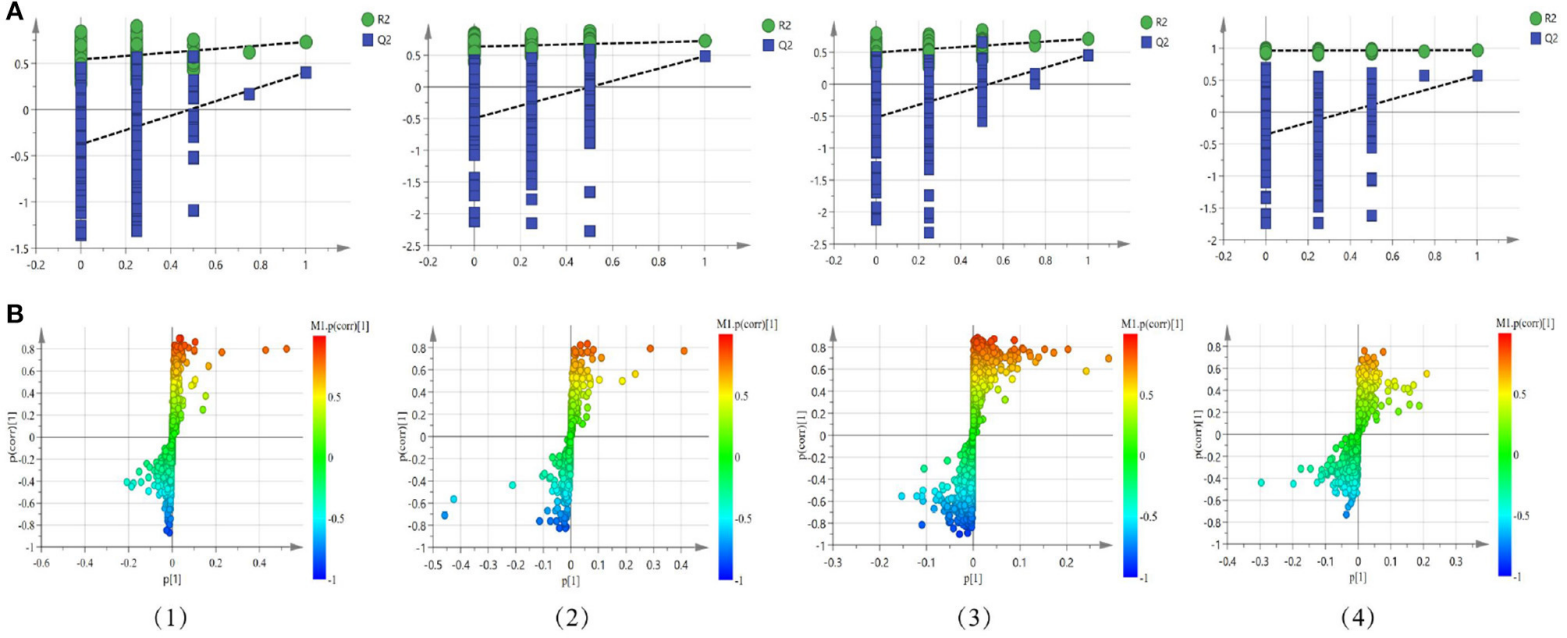
Permutation test plots **(A)** and S-plot **(B)** of OPLS-DA between the normal group and the model group in the positive mode of the non-polar part (1) and the positive mode of the polar part (2) of the serum, and in the positive mode of the non-polar part (3) and the positive mode of the polar part (4) of the cerebral cortex.

Compared with PCA, OPLS-DA is a supervisory model analysis method. The main parameters to judge the quality of the model are R^2^Y (the value represents the interpretation rate of the model) and Q^2^ (the value represents the prediction rate of the model). According to PCA, serum samples of the normal group and the model group were different to some extent. In order to obtain metabolite information leading to such significant differences, the supervisory multidimensional statistical method OPLS-DA was used to analyze the data, and the results are shown in Figure 5. In the serum sample, for the non-polar part, R^2^Y = 0.988 and Q^2^ = 0.898, and for the polar part, R^2^Y = 0.908 and Q^2^ = 0.686, in the cerebral cortex sample, for the non-polar part, R^2^Y = 0.729 and Q^2^ = 0.42; and the polar part, R^2^Y = 0.93and Q^2^ = 0.774, indicating that the model was established successfully. The permutation test plots and S-plot of OPLS-DA are shown in Figure 6.

### Identification of Potential Metabolites

In OPLS-DA, the potential biomarkers of metabolites were filtered by VIP > 1, excluding variables with large confidence intervals and connecting with the *P*-value of the peak area of the *t*-test (*P* < 0.05). In accordance with the above principle, 20 biomarkers and 12 biomarkers were, respectively, determined in the serum and the cerebral cortex. The detailed results are shown in Table 3. Metabolites displayed significant differences among the PRA, AF and PF groups and the model group. In the serum, PRA had an effect on 15 biomarkers (*P* < 0.05 or *P* < 0.01 or *P* < 0.001), AF had an effect on 11 biomarkers (*P* < 0.05 or *P* < 0.001), and PF had an effect on 10 biomarkers (*P* < 0.05 or *P* < 0.01). Meanwhile, in the cerebral cortex, LysoPE and another eight different metabolites were significantly reduced by PRA (*P* < 0.05 or *P* < 0.01 or *P* < 0.001). PF had significant effects on seven different metabolites (*P* < 0.05 or *P* < 0.01), and AF had similar effects to PF, significantly reducing the contents of seven different metabolites (*P* < 0.05 or *P* < 0.01). The above results indicate that the administration group demonstrated certain antidepressant effects.

**Table 3:** Potential biomarkers in the serum and cerebral cortex in the model rats.

**No**.	**VIP**	***m*/*z***	***t*** **_R_/min**	**Compound**	**Trend**				**Acquisition mode**	**Sample**
					**M**	**PRA**	**PF**	**AF**		
PM 1	1.00	780.553	10.361	PC (20:4/16:1)	↓^▴^	↑	↑	↑	ESI^+^(Nonpolar)	Serum
PM 2	1.14	792.588	10.641	PC (20:5/P-18:0)	↓^▴^	↑^**^	↑	↑	ESI^+^(Nonpolar)	
PM 3	1.07	772.621	12.870	PC (P-18:0/18:1)	↓^▴^	↑^**^	↑	↑	ESI^+^(Nonpolar)	
PM 4	1.15	996.800	16.993	TG (18:1/22:5/22:6)	↑^▴^	↓	↓	↓	ESI^+^(Nonpolar)	
PM 5	1.02	998.815	17.216	TG (18:1/22:6/22:4)	↑^▴▴^	↓	↓	↓	ESI^+^(Nonpolar)	
PM 6	2.69	688.602	17.666	CE (20:5)	↑^▴▴▴^	↓	↓	↓	ESI^+^(Nonpolar)	
PM 7	1.28	526.292	2.779	LysoPE (22:6/0:0)	↑^▴^	↓^*^	↓^*^	↑^*^	ESI^+^(Nonpolar)	
PM 8	1.01	572.371	3.960	LysoPC (22:4)	↓^▴^	↑^*^	↑^*^	↑^*^	ESI^+^(Nonpolar)	
PM 9	1.00	548.371	4.317	LysoPC (20:2)	↓^▴^	↑^*^	↑	↑	ESI^+^(Nonpolar)	
PM 10	2.42	508.376	4.571	LysoPC (P-18:0)	↓^▴▴▴^	↑^**^	↑	↑	ESI^+^(Nonpolar)	
PM 11	1.33	510.392	6.082	LysoP C (O-18:0)	↓^▴▴^	↑^*^	↑	↑^*^	ESI^+^(Nonpolar)	
PM 12	1.83	288.216	1.628	L-Octanoylcarnitine	↑^▴^	↓^*^	↓^*^	↓^*^	ESI^+^(Polar)	
PM 13	1.65	274.200	1.740	Heptanoylcarnitine	↑^▴▴^	↓^**^	↓^**^	↓^**^	ESI^+^(Polar)	
PM 14	1.57	232.154	2.203	Butyrylcarnitine	↑^▴^	↓^*^	↓^*^	↓^*^	ESI^+^(Polar)	
PM 15	1.39	209.091	4.713	l-Kynurenine	↓^▴^	↑^***^	↑	↑^*^	ESI^+^(Polar)	
PM 16	1.34	132.065	5.840	4-Hydroxyproline	↓^▴▴^	↑	↑^*^	↑^*^	ESI^+^(Polar)	
PM 17	1.20	130.049	6.259	Pyrrolidonecarboxylic	↓^▴^	↑^*^	↑^*^	↑^*^	ESI^+^(Polar)	
PM 18	1.19	147.076	6.259	L-Glutamine	↓^▴^	↑^*^	↑^*^	↑^*^	ESI^+^(Polar)	
PM 19	1.18	133.060	6.393	L-Asparagine	↓^▴^	↑^*^	↑^*^	↑	ESI^+^(Polar)	
PM 20	1.15	176.102	6.451	Citrulline	↓^▴^	↑^*^	↑^*^	↑^*^	ESI^+^(Polar)	
PM 1	1.00	780.553	10.361	PC (20:4/18:2)	↑^▴▴^	↓	↓	↓	ESI^+^(Nonpolar)	Cerebral cortex
PM 2	1.14	792.588	10.641	PC (22:6/14:0)	↑^▴^	↓	↓	↓^*^	ESI^+^(Nonpolar)	
PM 3	1.28	526.292	2.779	LysoPE (0:0/18:2)	↑^▴^	↓^*^	↓^*^	↓^**^	ESI^+^(Nonpolar)	
PM 4	1.01	572.371	3.960	LysoPE (22:6/0:0)	↑^▴^	↓	↓	↓	ESI^+^(Nonpolar)	
PM 5	1.00	548.371	4.317	LysoPE (18:2/0:0)	↑^▴^	↓^*^	↓^*^	↓^*^	ESI^+^(Nonpolar)	
PM 6	2.42	508.376	4.571	LysoPE (18:1/0:0)	↑^▴^	↓	↓	↓	ESI^+^(Nonpolar)	
PM 7	1.33	510.392	6.082	LysoPC (24:0)	↑^▴^	↓^**^	↓^*^	↓^*^	ESI^+^(Nonpolar)	
PM 8	1.83	288.216	1.628	Valerylcarnitine	↑^▴▴^	↓^**^	↓^**^	↓^**^	ESI^+^(Polar)	
PM 9	1.65	274.200	1.740	3-Hydroxyhexadecanoylcarnitine	↑^▴▴^	↓^*^	↓^*^	↓	ESI^+^(Polar)	
PM 10	1.57	232.154	2.203	Tiglylcarnitine	↑^▴▴^	↓^**^	↓^**^	↓^**^	ESI^+^(Polar)	
PM 11	1.39	209.091	4.713	L-Carnitine	↑^▴^	↓^*^	↓	↓	ESI^+^(Polar)	
PM 12	1.34	132.065	5.840	Butyrylcarnitine	↑^▴▴^	↓^***^	↓^**^	↓^**^	ESI^+^(Polar)	

^▴▴▴^ P < 0.001, compared with the normal group; *P < 0.05, **P < 0.01, ***P < 0.001, compared with the model group.

*“↑”Means the metabolite increased and “↓” means the metabolite decreased, compared with normal group or model group*.

*PC, phosphatidylcholine; TG, triglyceride; CE, cholesterol ester; LysoPE, lysophosphatidylethanolamine; LysoPC, lysophosphatidylcholines; M, model group; PRA, Aqueous extract of Paeoniae Radix Alba group; PF, paeoniflorin group; AF, albiflorin group*.

^▴▴^*p < 0.01, ^▴^p < 0.05, compared with the normal group*.

**Figure 7:**
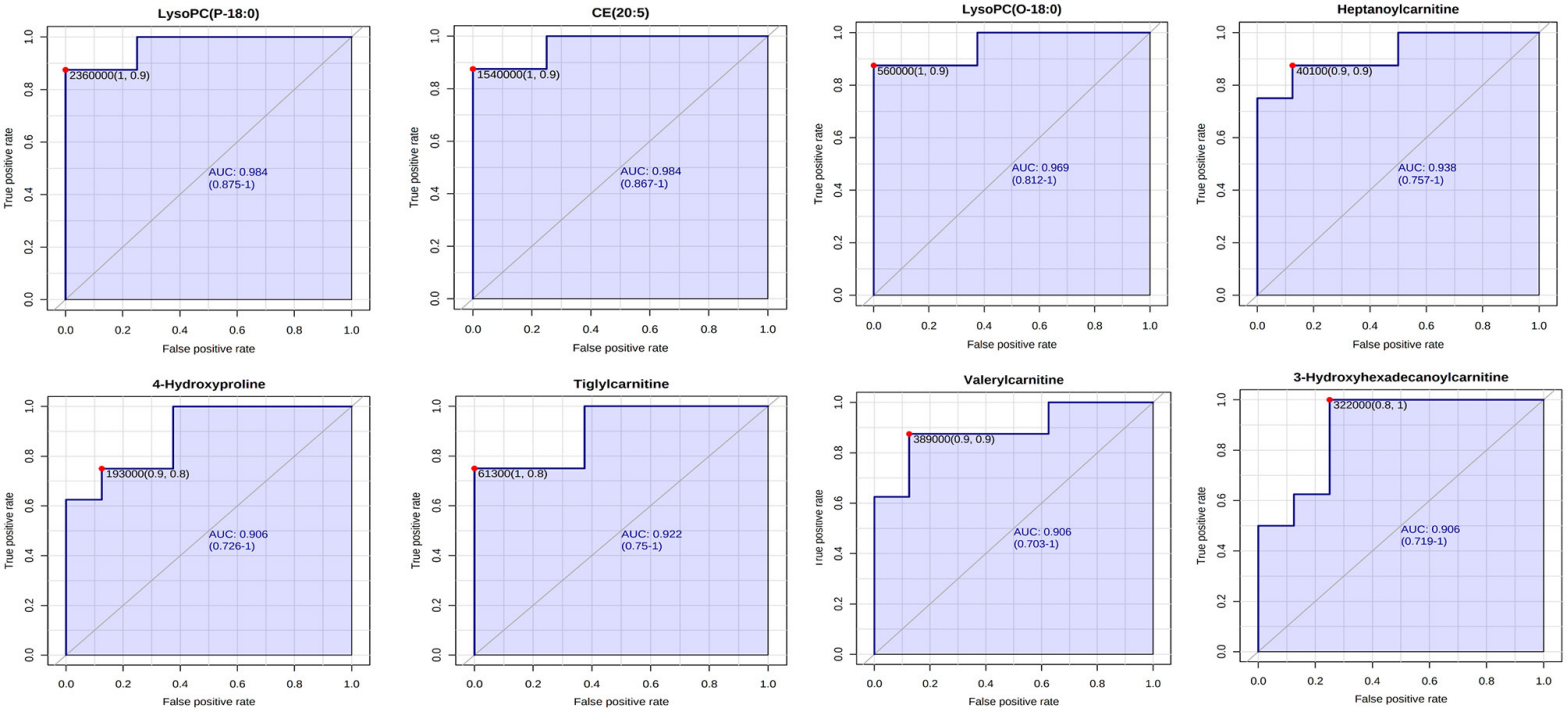
ROC analysis of potential biomarkers in the serum and cerebral cortex.

**Figure 8:**
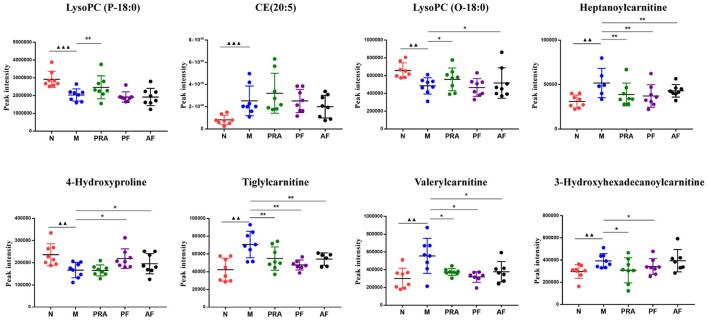
Potential biomarkers in the serum and cerebral cortex. ^▴▴▴^*P* < 0.001, compared with the normal group; *P < 0.05, **P < 0.01, compared with the model group. N, normal group; M, model group; PRA, Aqueous extract of *Paeoniae Radix* Alba group; PF, paeoniflorin group; AF, albiflorin group. ^▴▴^p < 0.01, compared with the normal group.

Receiver operating characteristic (ROC) analysis was used to further evaluate the reliability of potential biomarkers (30), with the area under the ROC curve (AUC) > 0.9 as the standard, eight differential metabolites were further screened, five in a serum sample and three in a cerebral cortex sample, which may become potential markers of AF and PF for antidepression effects. The detailed results are shown in Figures 7, 8.

### Metabolic Pathway Analysis

**Figure 9:**
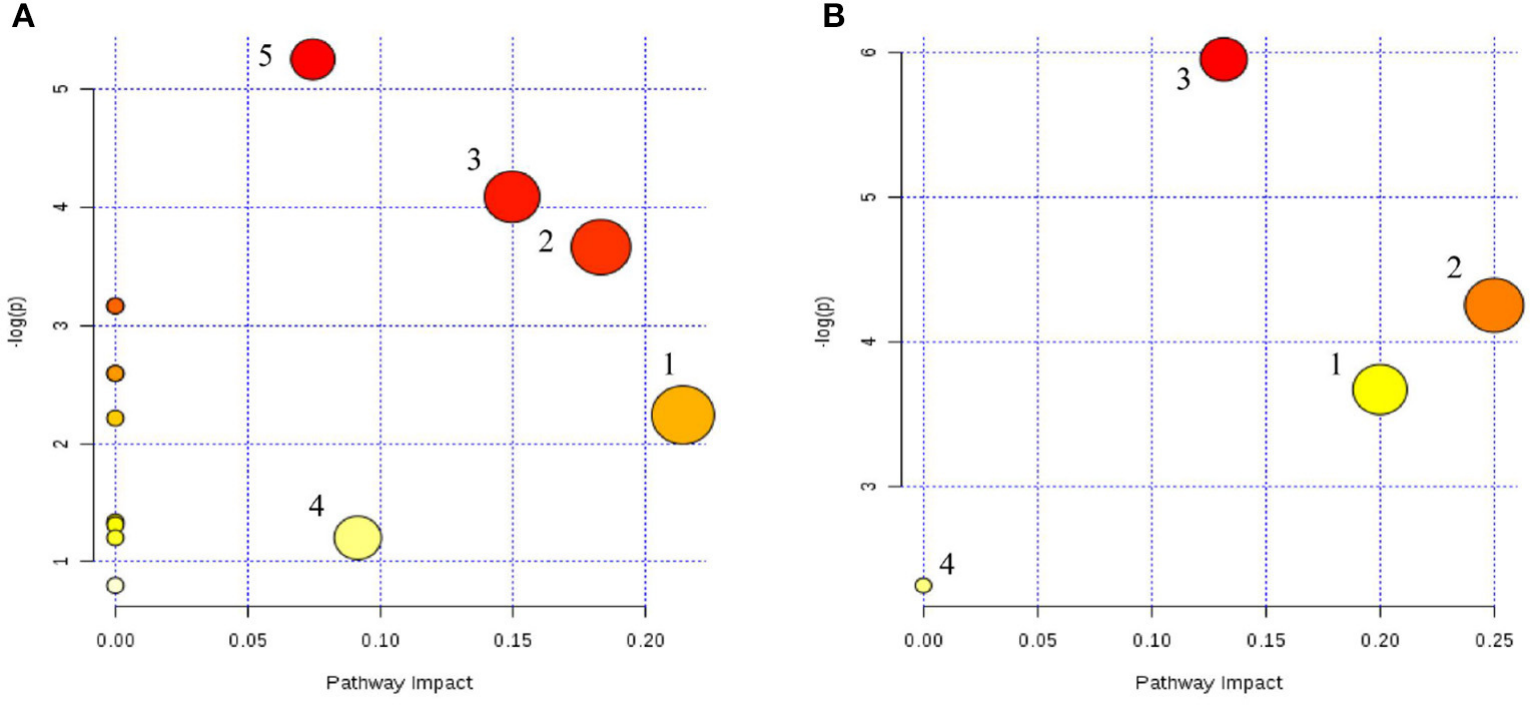
Serum **(A)** and cerebral cortex **(B)** differential metabolite-related metabolic pathways. **(A)** (1) Lipid metabolism; (2) glycerophospholipid metabolism; (3) alanine, aspartate, and glutamate metabolism; (4) tryptophan metabolism; and (5) arginine and proline metabolism. **(B)** (1) Linoleic acid metabolism; (2) alpha-linoleic acid metabolism; (3) glycerophospholipid metabolism; (4) arachidonic acid metabolism.

Metabolic pathway analyses were performed with MetaboAnalyst 4.0, an easy-to-use web-based tool suite (31). As shown in Figure 9, the metabolic pathways of serum were ether lipid metabolism; glycerophospholipid metabolism; alanine, aspartate, and glutamate metabolism; tryptophan metabolism; and arginine and proline metabolism, while in the cerebral cortex, the metabolic pathways were linoleic acid metabolism; alpha-linoleic acid metabolism; glycerophospholipid metabolism; and arachidonic acid metabolism.

The same metabolic pathway was seen in two samples, which may reflect the characteristics of multi-target drug action.

### Correlation Analysis Between Potential Biomarkers and Biochemical Indicators

**Figure 10:**
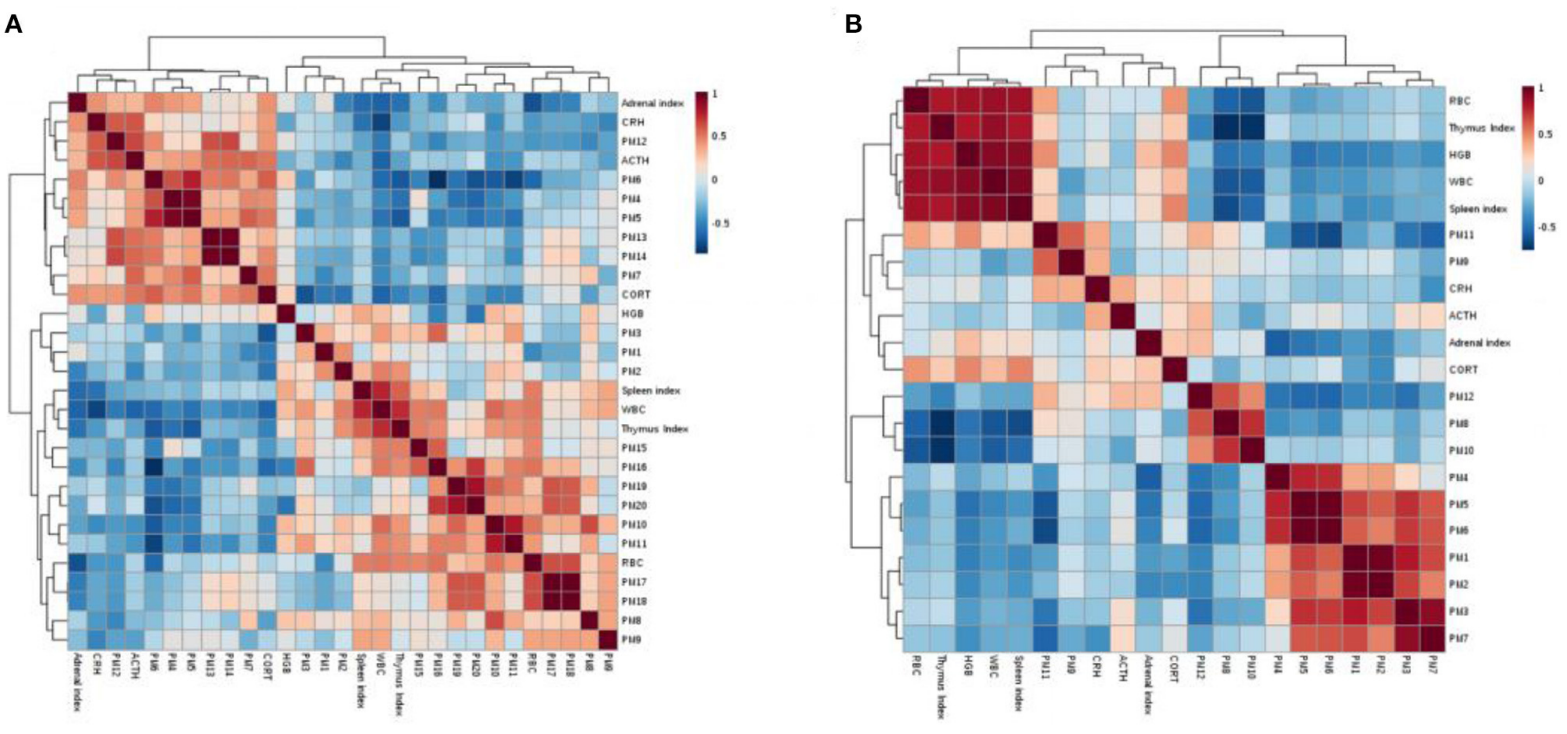
Serum **(A)** and cerebral cortex **(B)** correlation analysis between potential biomarkers and biochemical indices.

Correlation analysis can reflect the relationship between different metabolites in metabolic pathways. The Pearson correlation analysis based on the MetaboAnalyst website (http://www.metaboanalyst.ca/) was used to screen the correlation analysis between 20 different metabolites in the serum, 12 different metabolites in the cerebral cortex and indicators of WBC, RBC, HGB, ACTH, CORT, CRH, the thymus index, spleen index and adrenal index. *r* > 0.5 or *r* < −0.5 is considered to be correlated with each other, and the results are shown in Figure 10. The colors in the figure represent the correlation values between different metabolites, with red representing a positive correlation and blue representing a negative correlation.

There is a certain correlation between potential biomarkers, between potential biomarkers and biochemical indicators, and between biochemical indicators. For example, in the correlation analysis of biomarkers and biochemical indicators in the cerebral cortex, WBC, RBC, the thymus index and the spleen index are negatively correlated with valerylcarnitine and tiglycarnitine (*r* = −0.598; −0.569; −0.552; −0.5597;−0.738; −0.722; −00.635; and −0.569, respectively); these correlation results will help to better reveal the antidepression mechanism of drugs.

## Discussion

Depression is a persistent and recurring mental illness, perceived as a multifactorial condition that involves neural, endocrine, and immune pathways (32). The Global Burden of Disease Study 2019 showed that depression has become one of the 10 major diseases contributing to the increased Global Burden of Disease, and the sixth reason affecting the disability adjusted life years (33). Patients undergoing and post radiotherapy with comorbid depression are negatively affected, demonstrating a decline in their of quality of life, an increase in medical costs, the limitation of their treatment course and recovery, and so on (34). These patients have to endure the double pain of cancer and depression, and they urgently need to receive more attentions and effective interventions.

Due to its few adverse effects, multi-mechanisms and multi-targets, traditional Chinese medicine is considered as a valuable reservoir for the development of novel antidepressant and anticancer drugs (35, 36). *Paeoniae Radix* Alba (PRA) is a traditional Chinese medicine that exhibits the unambiguous effects of nourishing the blood and smoothing the liver, and is frequently used for treating diseases with liver-qi stagnation and blood-deficiency syndrome (LSBD) in China (37). Cancer radiotherapy patients often have depression (38), fatigue (39) and malnutrition (40) symptoms, and according to TCM theories, they can be categorized into LSBD syndrome. Therefore, on the one hand, patients need to regulate their emotions to alleviate depressive symptoms, and they also need to tonify their qi and blood to enhance immunity. We therefore provide an illustrative case to show whether albiflorin (AF) and paeoniflorin (PF), extracted from PRA, had antidepression effects and to explore their mechanisms.

An appropriate animal model is crucial for unraveling the effects and mechanisms of medicine. Unlike usual depression, cancer-related depression has a defect in immune function, so both depression and immunocompromised states need to be considered in the modeling. Therefore, considering the broad spectrum and multiple factors of cancer-related depression and the treatment of cancer, we explored the rat model and successfully established the model by combining irradiation with chronic restraint stress and solitary confinement (28), in a multiple stress reaction, which can simulate the state of cancer-related depression patients to a certain extent, such as decreased peripheral blood count, decreased immunity and a series of depressive symptoms. From the perspective of TCM, the model rats not only present the endocrine-immune system disorders caused by the deficiency of yin blood in the liver meridian, but also show changes in the neuroendocrine system disorders caused by the stagnation of liver qi. This is also consistent with the view that the pathogenesis of depression is involved in the neuroendocrine-immune network (41, 42). In this paper, pharmacological studies showed that depression-like behavioral deficits in model groups and the lowered or increased levels of the peripheral hemogram, HPA axis, and organ index were ameliorated by AF and PF. Metabonomics research found 20 and 12 biomarkers in the serum and cerebral cortex of the model, respectively, and eight potential biomarkers were further screened in ROC analysis. AF and PF can regulate several of these metabolites, involving multiple metabolic pathways, and there was no significant difference in the regulatory effects of AF and PF, which may be due to the relatively similar chemical structure between the two.

There is increasing evidence that the neuroendocrine immune system is closely related to the occurrence of depression (41). The immune system affects the secretion of neurotransmitters in the nervous system and peptide hormones in the endocrine system through cytokines and hormones secreted by immune cells. On the contrary, immune cells can express a variety of neurotransmitters and hormone receptors, neurotransmitters and hormones produced by the neuroendocrine system that are used in immune cells. Through the interaction of these small signal molecules, the neuroendocrine immune network is formed. Peripheral blood and organ indexes are important immune indexes, which are closely related to the function of the immune system (43). Red blood cells, white blood cells and hemoglobin are the most important cell components in the blood, white blood cell count is an easy marker for the estimation of immune function (44). Studies have found that anemia or abnormal erythrocyte parameters such as red blood cell count, hemoglobin in patients with depression and changes in erythrocyte may be associated with the risk of depression (45, 46). While the thymus and spleen are important immune organs in the body, the organ indexes can be used to evaluate the immunity of organisms (47). In this experiment, the red blood cell count, white blood cell count, hemoglobin and the thymus index and the spleen index of rats in the model group were significantly decreased, indicating that the body had abnormal immune system function, which was consistent with the literature reports. AF and PF could significantly increase the thymus index and spleen index, improving the abnormal immune function of the body. With increasing blood–brain barrier permeability, peripheral cytokines can be actively transported into the CNS, subsequently, followed by a reduction in serotonin neurotransmission and the activation of the HPA axis (41). As the hub of the neuroendocrine network, the HPA axis plays an important role in the pathogenesis of depression, with the dysregulation of the HPA axis (48), increasing extracellular glutamate levels and glutamate neurotransmission, and then impacting hippocampal neurogenesis, and through glucocorticoids, the HPA axis is involved in anti-inflammatory, pro-inflammatory and other immune regulatory processes (49). In major depressive disorder, the glucocorticoid receptors' sensitivity decreased, leading to the reduction in the HPA axis' negative feedback mechanism to maintain homeostasis, which leads to the hypersecretion of CRH, ACTH and cortisol (50). In mice with breast cancer concurrent with depression, the content of the HPA axis, such as CORT, ACTH, and CRH, were increased (51). Our study results are consistent with the literature which indicates that the model rats were in a state of depression, and paeoniflorin and albiflorin could play an antidepressant role by regulating the HPA axis. Sucrose preference, open field test and elevated plus-maze are classic indicators used to evaluate animal depression or anxiety (52, 53). In this study, various behavioral traits of model rats were significantly changed, suggesting that the rats were in a state of depression. AF and PF can effectively alleviate this state. Thus, pharmacological studies have shown that AF and PF have antidepression effects, the primary mechanism of which may be related to the regulation of neuroendocrine immune system abnormalities.

Metabonomics exhibits an impressive and ever-increasing coverage of endogenous compounds, focuses on the holistic investigation of multi-parametric metabolite responses of living systems, which is consistent with the holistic view of TCM and the multi-mechanism and multi-target characteristics of TCM and has been widely applied on TCM research (54, 55). We applied the metabonomics method to research the changes in serum and cerebral cortex metabolites, and to explore the action mechanism of drugs. The results showed that multiple metabolites and pathways were involved.

Ether esters have a certain influence on the permeability and fluidity of cell membranes, and are also involved in intercellular signal transduction, antioxidant scavenging, oxidative stress reactions and other processes (56). Abnormal secretion of ether esters is found in mental illness, like Alzheimer's disease (57). Phosphatidylcholine (PC) is the most abundant phospholipid in membrane bilayers. PC has anti-inflammatory effects, and can regulate systemic inflammation through the brain–gut axis (58). Lysophosphatidylcholines (LPC) is produced by the hydrolysis of PC catalyzed by phosphatase A, which also has important roles in membrane permeability and fluidity. Previous studies suggested that chronic unpredictable stress mice and mice treated with antidepressant drugs shown abnormal phospholipid contents (59, 60), which was also confirmed in several clinical studies, and some scholars believe that lipids are an effective target for preventive medicine of psychiatric disorders (61). In our study, PC and LPC as potential biomarkers were involved in ether ester and glycerolphospholipid metabolism, and the content of PC and LysoPC significantly decreased in model rats, while AF and PF can regulate this abnormality, affecting the metabolism of ester and glycerol phospholipid, playing a role in antidepression. However, one point should be noted— during the screening of differences, the contents of the metabolites phosphatidylcholine and lysophosphatidylcholine decreased in the serum of model rats, but increased in the cerebral cortex. This result suggests that the pathogenesis of the disease and the mechanism of action of drugs may have different effects in different biological samples, and also verifies the functional characteristics of multi-component and multi-target of TCM.

Glutamine has a certain effect on the nervous system and immune system (62, 63). One study found that CNS glutamate regulation and the antidepressant effect maybe influenced by inhibiting glutamate release by peripheral cancer cells (64). Clinical studies have also shown that patients with major depressive disorder had abnormal glutamate and glutamine levels (65), and glutamate can cause HPA axis hyperactivity (66). At the same time, the glutamine blockade can overcome tumor immune evasion (67); this is consistent with the results of pharmacological studies. Asparagine is converted from aspartic acid and plays an important role in the pathogenesis of depression (68), asparagine endopeptidase cleaves protein substrates on the C-terminal side of asparagine, and deletion of it can reduce anxiety- and depressive-like behaviors in mice (69). Our study results show that the contents of glutamine and asparagine in the model group were decreased, indicating that the model rats were in a certain state of depression, with abnormal metabolism of alanine, aspartic acid and glutamic acid, and AF and PF could increase their contents and play a therapeutic role.

Kynurenin is an important substance in tryptophan metabolism, and a reduction in its content will lead to abnormal tryptophan metabolism. More and more studies have found that tryptophan-kynurenin metabolism plays an important role in depression, which is manifested as the abnormal secretion of tryptophan-kynurenin (70, 71). Tryptophan metabolism also closely associated with inflammation, TNF-α and other inflammatory factors, can activate 2,3-dioxygenase oxygen (IDO) and tryptophan enzyme (TDO) through inflammatory signaling pathways, increase tryptophan decomposition and reduce its peripheral content. At the same time, this inflammatory factor can cause the brain tryptophan metabolism to increase when its content is lower. Finally, it can lead to abnormal urinary acid and its related metabolic pathways (72). Tryptophan is also the precursor of serotonin (5-HT). The abnormal metabolism of tryptophan causes the abnormal metabolism of 5-HT neurotransmitters, manifested as insufficient synthesis and transmission disorder, and is closely related to the pathogenesis of depression (73), which is consistent with the previous research results of our research group (16). In this experiment, the content of kynurenine in the model rats decreased, indicating that the rats showed depression-like behavior and abnormal tryptophan metabolism. AF and PF could affect tryptophan metabolism by increasing the content of kynurenine.

The potential biomarkers Glutamine, Citrulline and 4-hydroxyproline are involved in arginine and proline metabolism. Citrulline is phosphorylated by ornithine and carbamyl, and is also an arginine metabolite, which is one of the core reactions of the urea cycle, and participates in the NO metabolic pathway, which is under the catalysis of endothelial nitric oxide synthase (eNOS), Meanwhile, arginine combines with oxygen to produce NO, and L-citrulline is the end product of NO production (74). The related literatures has reported that the L-arginine and L-citrulline concentrations were significantly lower in severe depression patients than in healthy controls, which is a possible explanation for the decrease in NO metabolites in depression patients (74). At the same time, in stress-loaded senescence-accelerated mice, L-arginine reduces oxidative damage and enhances mitochondrial functions in the brain and exerts excellent anti-stress effects (75), and abnormal metabolism of arginine and proline metabolism in rats and patients with depression (76, 77). In our research, the contents of glutamine, citrulline and 4-hydroxyproline in the serum of model rats were significantly decreased, which was consistent with the literature reports. AF and PF increased their contents, and then affected the metabolism of arginine and proline.

Carnitine is an amino acid derivative, synthesized from the essential amino acids methionine and lysine. It plays an important role in the oxidative stress, fat metabolism and energy metabolism of the body, and is involved in the synthesis of fat, the transportation and oxidation of fatty acids, and the utilization of ketone bodies (78). The increase in carnitine content can reduce the content of acetyl-CoA and fatty acids in the body; fatty acids are the precursors of active substances such as prostaglandins, and participate in the process of inflammation and immunity in the body. Carnitine also can prevent the programmed cell death of immune cells (79). The decrease in their content will affect the process of immune regulation in the body. As the short-chain ester of carnitine, acetyl-L-carnitine (ALC) has received more attention in antidepression, including mediating the PI3K/AKT/BDNF/VGF signaling pathway (80), and brain energy metabolism (81). ALC is considered a promising antidepressant, especially for older patients (82, 83). Under the action of carnitine acyltransferase *in vivo*, L-carnitine uses acylcarnitine as a carrier to deliver short-chain acyl groups in the mitochondria to the outer mitochondrial cell membrane, and transfer the long-chain fatty acids outside the membrane to the membrane, so that they can perform β-oxidation in the mitochondrial matrix to produce energy (84, 85), and maintain the balance of energy metabolism in the body. The decrease in carnitine content will increase the concentration of acetyl-CoA in the mitochondria. Meanwhile, the fatty acids outside the mitochondrial cell membrane cannot enter the cell membrane, which affects the Krebs cycle. In this experiment, the contents of L-octylcarnitine and other carnitines in the serum and L-carnitine, butyrylcarnitine and other carnitines in the cerebral cortex of model rats increased, suggesting that the bodies of model rats demonstrated β-abnormal oxidative function and an imbalance of energy metabolism, which are consistent with the literature reports, indicating that the model rats are in a certain state of depression. AF and PF can reduce the content of carnitine metabolites, regulate the body's energy metabolism, and exert antidepressive effects.

Glycerol phospholipids can be decomposed to form hemolytic lecithin and arachidonic acid under the action of phospholipase, and the change in glycerol phospholipid content can lead to the change in arachidonic acid content *in vivo* (81). Arachidonic acid is a kind of unsaturated fatty acid, that can be in the ring oxidase, and it can undergo lipid oxygenation under the action of enzymes to a variety of important bioactive substances such as leukotrienes and prostaglandins, which help to increase vascular elasticity, reduce blood viscosity, and regulate blood's physiological activity, such as in the body's inflammatory response and immune response processes (86, 87). Arachidonic acid plays an important role in brain diseases and depression—derivatives of arachidonic acid can increase dopamine release by activating the cannabinoid type-1 receptor (88, 89). The pharmacological research results of this study show that the metabolism of relevant indexes of peripheral hemogram is abnormal, and the function of blood cells is reduced in model rats. The metabonomic research results show that the content of phosphatidylcholine in the brain tissue of model rats is increased, indicating that there is an abnormal metabolism of arachidonic acid in the body, which impairs the relevant functions of the immune system. AF and PF can play an antidepressant role by reducing the content of phosphatidylcholine and then affecting the metabolism of arachidonic acid.

## Conclusions

**Figure 11:**
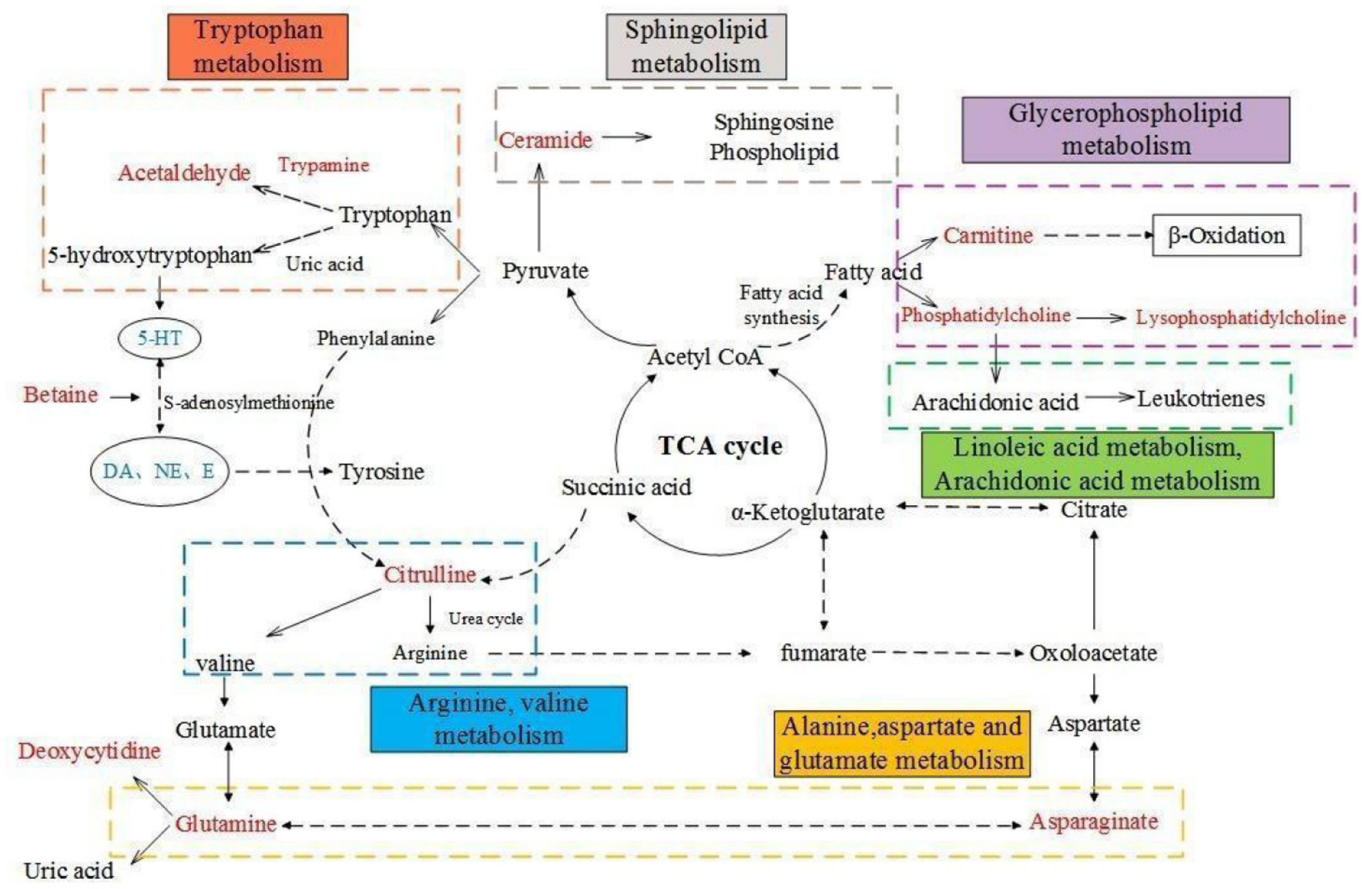
Overall metabolic pathway map of model rats. Red characters are metabolites for screening, blue are pharmacodynamic index; Solid line is one-step reaction, dotted line is multi-step reaction.

The present studies suggest that AF and PF caused a significant improvement in the pharmacodynamic index by modulating the neuroendocrine-immune network and regulating abnormal metabolic pathways (Figure 11) and demonstrate that the metabolites may be useful as potential markers for diagnosing and monitoring cancer-related depression. These findings may supply beneficial hints for the treatment of cancer-related depression, and thus deserve further clinical investigations.

## Data Availability Statement

The original contributions presented in the study are included in the article/supplementary material, further inquiries can be directed to the corresponding authors.

## Ethics Statement

The animal study was reviewed and approved by Experimental Animal Ethics Committee of Beijing University of Chinese Medicine.

## Author Contributions

Experiments designed and supervised: LW and JZ. Experiments conducted: DZ, NY, and CW. Resources and formal analysis: CH. Original manuscript wrote: DZ. Manuscript reviewed and edited: YZ, WF, and JZ. All authors have read and agreed to the published version of the manuscript.

## Funding

This work was supported by the National Natural Science Foundation of China (81473370) and National Key R&D Program of China (2018YFC1706800).

## Conflict of Interest

The authors declare that the research was conducted in the absence of any commercial or financial relationships that could be construed as a potential conflictof interest.

## Publisher's Note

All claims expressed in this article are solely those of the authors and do not necessarily represent those of their affiliated organizations, or those of the publisher, the editors and the reviewers. Any product that may be evaluated in this article, or claim that may be made by its manufacturer, is not guaranteed or endorsed by the publisher.
